# Co-creating a knowledge base in the “22q11.2 deletion syndrome” community

**DOI:** 10.1007/s12687-019-00425-8

**Published:** 2019-05-25

**Authors:** Roberta Rizzo, Marianne Van den Bree, Aimee Challenger, Andrew Cuthbert, Michael Arribas Ayllon, Angus Clarke, Rose Thompson

**Affiliations:** 1 Cardiff University, Cardiff, UK; 2 The McPin Foundation, London, UK

## Abstract

22q11.2 DS is characterised by its variability, rarity and variety of features ranging from congenital heart conditions to psychiatric and behavioural issues. As a result, health information–seeking behaviour is different from other more common conditions. An exploratory study was carried out to understand how parents access information and support, and how that information is shared. Qualitative interviews were carried out with families and support group representatives, and thematic analysis was applied. Four main themes emerged from our findings: perceptions of clinical expertise, parent empowerment, support group activities and community building via an Internet platform. Our thematic analysis enabled the construction of a possible model of information-seeking behaviour in parents and carers of children with 22q11.2 DS. We discuss the model and how the understanding of how information is shared and gathered can aid in clinical practice.

## Introduction

The 22q11.2 deletion syndrome (22q11.2 DS) is a chromosomal disorder (1:1000 births reported). The phenotype has been described as being of variable pattern which includes aspects of physical, developmental and mental health (Shprintzen 2008). Some researchers suggest that the syndrome is clinically under-recognised (McDonald-McGinn 2013, Shprintzen 2008, Oskarsdottir 2004) because variability is a major obstacle for diagnosis and long-term management. Thus, due to the complexity of 22q11.2 DS, it is often difficult for parents or carers to understand the condition or to receive the right support before or after diagnosis (Dimond 2010).

In an extensive review, Pelentsov et al. (2016) suggested that the care needs of families affected by rare diseases in general are not met by health care professionals. The main areas identified the range from emotional and psychological needs to practical and social needs. These shortfalls in the provision of support to families have been reiterated by Rare Disease UK in their recent documentation (Limb and Nutt 2011).

Similar to other rare conditions, information about 22q11.2 DS has become more available recently with advances in research and information technologies. For instance, the Internet has become a powerful tool for parents of children with rare conditions to seek information and relevant support. It also plays a major role in the way parents make decisions about caring for a child with 22q11.2 DS (Van den Bree et al. 2013). At present, it is now possible to find and access specialist support groups and forums which relate to a variety of health conditions; many of these have been described approvingly by parents (Gunderson 2011).

The Internet has also developed into a social platform that facilitates connections between families with similar experiences (Oprescu et al. 2013, Gunderson 2011, Plumridge et al. 2010). Organised support groups use the Internet as a digital medium to create communities of support for affected families. In addition to providing emotional and practical support, these communities also function to create hubs of information- and awareness-raising activities. Although the Internet is being increasingly regarded as a helpful resource for families, there are consistent concerns from both support group facilitators and professionals that information may be inaccurate or overwhelming (Ziebland and Wyke 2012, Waldron et al. 2011).

Patient organisations and support groups have evolved in recent years as essential producers of knowledge across the social, scientific and political spectrum. Carlos Novas calls this phenomenon ‘the political economy of hope’, which he describes as being:

a mutation in the biopolitics of our present whereby persons affected by genetic conditions have become significant authorities who are engaged in the promotion of the health and wellbeing of individuals and populations, who directly contribute to the production of biomedical knowledge. (Novas, 2006 p.290)

In line with these recent developments, the present study explores how families caring for someone with 22q11.2 DS produce and share information. The primary aim is to explore how online communities of support are shaping the experiences of families’ offline. A secondary aim is to understand how support groups facilitate information sharing and handle the dynamics and politics of their particular role as advocates and information providers. In what follows, we examine the ways in which online communities bring together families, support groups and clinicians. By evaluating these processes, we show how online practices are co-creating knowledge and support for families coping with 22q11.2 DS.

## Methods

The present study forms part of a larger project titled ‘Improving services for children with chromosome disorders’, the purpose of which is to explore the experiences of families with different CNVs (Copy Number Variants, e.g. chromosomal re-arrangements, such as 22q11.2 deletion). This study was carried out by the ECHO (Experiences of CHildren with cOpy number variants) research group in Cardiff, which explores the phenotypic outcomes and developmental pathways of people with CNVs. A large part of the study assesses the cognitive, behavioural and psychological functioning of children and adults diagnosed with these conditions (Niarchou et al. 2014; Chawner et al. 2017).

The current paper explores social issues around online resources, support groups and parental support. A mixed methods approach was used, including the collection of survey data and interviews with parents of children with 22q11.2 DS and representatives of support groups. This paper will present data from the qualitative component of the ECHO project.

The principal aim of the qualitative study was to explore the following issues in relation to information and support after receiving a diagnosis of a copy number variant:

1. Experiences of diagnosis
2. Sources of information accessed by respondents
3. Perceived helpfulness and usability of sources of information

In the online survey, a question was included asking respondents to indicate whether they would be willing to participate in a subsequent research interview. Among those who did, respondents were contacted via phone or e-mail after which an appropriate time and date for an interview was arranged. Face-to-face, telephone or Skype interviews were conducted to suit the availability and preferences of the participants. An information sheet about for the qualitative interview was sent along with a consent form prior to the interview. The interviews were semi-structured and participant-led, allowing participants to raise their own experiences and concerns.

Support group representatives were recruited by contacting relevant organisations in the UK. These include Max Appeal, Unique, 22q Crew and 22q Ireland. Interviews were arranged with consenting participants, who were asked about the aims and provision of support as well as the organisation’s history. Parts of the interview were dedicated to understanding the role of online social media in their provision of support.

All the interviews were transcribed verbatim by the lead researcher with names of people and places replaced with pseudonyms. Ethics approval for this study was obtained from Cardiff University’s School of Medicine Research Ethics Committee. Informed consent was obtained from all individual participants included in the study.

## Analysis

Interviews with parents and support group representative were analysed using an analytic method called ‘Thematic Analysis’ (Braun and Clarke 2006). A ‘theme’ is defined as patterned responses within the data set that are meaningfully related to a research question. Thematic analysis is a flexible, inductive approach of developing inferences that, although informed by a relevant literature, represent prevalent or important patterns within the data. Interview transcripts were coded at first provisionally and then gradually refined through a process of iterative reading and interpretation. Codes were reviewed by a second researcher to check the validity and clarity of categories and to remove any redundancies. Codes were then formally grouped into common themes relating to the research questions and were reviewed by the other authors.

## Participants

One hundred and fifty-one responses were collected from the quantitative survey in total. The replies were filtered to focus on those experiences of the 22q11.2 deletion copy number variant. Of those, 29 responses indicated that their child had 22q11.2 deletion syndrome (DS).

These responses will not be discussed in this current paper but a basis for organising and structuring the questions for the face-to-face interviews are reported here.

Of these 29 responses to the survey, 21 participants indicated that they were interested in taking part in a research interview and provided contact details. From these, 7 interviews were carried out with carers of children with 22q11.2 DS by the lead researcher. One interview was carried out by A.D. All eight interviews were carried out with mothers. Six interviewees were from the UK and Europe and the remaining two were from the USA. In all situations, the condition had raised de novo in the children.

Seven support group representatives were interviewed from four different UK support groups. These included two males and five females, four of whom cared for someone with 22q1121q11.2 DS.

## Findings

### Thematic analysis of interviews with parental and support group representatives

Seventy codes were initially generated in the first phase of data analysis. After revising the codes that met the validity of the research questions, forty-five codes where generated. After removal of seven synonymous codes, 38 codes were clustered into four related themes, with some codes overlapping more than one theme (see Table 1).

**Table 1:** Themes and code clusters

Themes	Codes
Perceptions of clinical expertise	• Complexity of condition • Complex terminology • Medical abandonment • Clinical inexperience due to rarity of condition • Communication • Emotional impact of diagnosis disregarded in clinical setting • Patchy access to services • Parental perception of blame by professionals
Parent empowerment	• Seeking answers • Proactively finding solutions • Sharing knowledge • Internet as information source • Internet as platform to share information • Community building • Seeking and sharing practical information • Positivity • Helping others
Support group activities	• Finding the right support • Support group duties/roles • Eliminating isolation • Raising awareness • Social media • Helping others • Community building • Personal experience • Conferences • Using the Internet • Bridge between families and clinicians
Community building via an online platform	• Vetting information • Online community • Sharing experiences • Information • Free access to information • Accessible information • Shrinking the world • Eliminating isolation • Learning from the experiences of older children of other parents • Seeking like families with same diagnosis

### Perceptions of clinical expertise

This theme reflects the ways in which parents perceive the care they received from various clinicians after receiving a diagnosis.

Respondents reported a lack of specific medical experience and knowledge regarding the condition. The ‘rarity’ of the condition appeared to ‘cloud’ major clinical consultations. For the most part, parents reported feelings of ‘abandonment’ by clinical services, and difficulties in accessing appropriate care and information. Two parents described encounters in which medical professionals did not know what to call the condition and could not establish a coherent relationship between the child’s symptoms. This was particularly noticeable among parents who received a diagnosis in the mid-1990s.

it was 15 years ago and at that point it was pretty much a new ha I’m doing new with the fingers now (R laughs) condition emm you know they were still consolidating I suppose. I assume that VCFS and 22q and di George were in effect the same condition, so there was emm (pause) I assume not a lot of information around at that point. And in particular a lot of doctors didn’t know about it emm but we weren’t told anything because I assume nobody knew the name for it. (Charlotte)

Charlotte describes the difficulty of receiving a conclusive diagnosis (and prognosis) for a child. The various names for the condition (‘VCFS’, ‘di George’) convey a sense of confusion among the medical profession. Her sarcasm (‘new ha I’m doing new with the fingers now’) implies that the medical staff were ignorant of the condition, which clashed with the conventional idea of doctors being all-knowing. But Charlotte acknowledges that medical knowledge was undergoing a period of ‘consolidation’, meaning that a genetic diagnosis was beginning to underpin the condition.

Support groups report that the main reason they are approached is because carers are dissatisfied with the information and support they receive from the medical profession. Some feel dismissed by medical professionals for asking questions about their child’s condition, while others describe being blamed and criticised by professionals.

Very often their parenting skills are being undermined by medical professionals… Because […] the dentist said: you’re not looking after your child’s teeth. And it’s got nothing to do with the mothers looking after the child’s teeth. It’s the hypoplasia, and the enamel and the oral stuff that’s going on with 22q. but if you don’t have that information as a parent you are going to come out of that dentist office, distraught and doubting yourself and your parenting skills and everything else, you know? (Support group representative D)

A majority of the parents referred to their experience of diagnosis as uniformly devastating and shocking. Although parents may have been aware of their child’s symptoms and complications, the moment of clinical diagnosis was usually described as overwhelming. Furthermore, the quality of information they received was uneven. Some experienced a sense of information overload while others described receiving insufficient information. In the following, a mother gives an extreme account of poor clinical communication.

I had Katy at 38 weeks, she stopped growing at 32, so they were concerned during my labour so they induced me – it wasn’t until she was 12 hours old that we went to [name] Hospital, and there they tested for 22q though nobody told us. We received it… written on a piece of paper before she was due to go in to theatre. When she was three weeks old. […] They gave us a piece of paper [with the diagnosis] and said if you would like to find out more please go to the library and google it. And that was all we were told! […] oh it was devastating. (Marianna)

There are many aspects of this account that describe a series of failures in diagnosis and communication. Marianna reports that she was unaware that her child was tested for a genetic disorder, the results of which are given before her child is about to go to surgery. There is no verbal communication or counselling but a written diagnosis (‘on a piece of paper’). Even though medical professionals often caution against parents using the Internet to seek medical information, Marianna reports being told to ‘google’ the information in a library. Accounts like these invoke a sense of abandonment by the medical profession.

Parents coping with a diagnosis of a rare condition, like 22q11.2 DS, felt they needed more than information. In many cases, psychological support was absent. Even the experience of seeing a ‘genetic counsellor’ highlighted a mismatch of expectations.

…still to this day, there is very little what I would call psychological counselling… genetic counselling is a little bit of a misnomer because people expect a counsellor. Emm which they don’t get. There is not enough psychological support, you know psychological support around a diagnosis or around having a child with an emm rare condition and learning disabilities (Amy)

Amy explains that ‘genetic counselling’ gives an unrealistic impression that people will receive psychological support but then these expectations aren’t met. She implies that families need not only emotional support to understand a genetic diagnosis but also support regarding the consequences of caring for a child with a ‘rare condition and learning disabilities’.

Although some parents conceded that medical professionals were genuinely ignorant of 22q11.2 DS, the poor communication and lack of support led parents to lose trust in clinical services, prompting them to explore their own pathways for seeking information and support.

### Support groups

Support groups represent a broad category that is heterogeneous and takes a variety of forms. Some groups are ‘formal’ in the sense that they are embedded within charitable, voluntary organisations and offer ‘top-down’ services for affected families. Other groups are ‘informal’ and community-based, offering a range of ‘bottom up’ services mainly through online forums. In this section, we focus on the structured activities of formal organisations that operate a website and hold organised events and conferences, but towards the end, expand on the informal support groups briefly.

Formal support groups are registered, charitable organisations often led by parents who have first-hand experience of living with an affected child. One representative described her own experience of receiving her child’s diagnosis of 22q.11.2 DS as follows:

When you have a child with a rare chromosome disorder and you’ve been given that diagnosis, it’s horrendous, no matter how much knowledge you had before, what a chromosome is or whatever, umm, it’s a real, physical pain you go through, a feeling of bereavement frankly, umm. You do not have the child you anticipated, you feel isolated and lost in this world of people with perfect children, all you can see is your, your supposedly imperfect child. It’s a very lonely place to be, it’s very sad (Support Group Representative C).

In addition to the shock of receiving a genetic diagnosis, a common theme among families is bereavement and isolation of not having a normal, healthy child. The kind of support families need to reduce this isolation is ‘information’, though not the kind of ‘chromosomal’ information provided by professionals. The respondent explains that ‘very often professionals don’t have the sort of information that we can give them’. Since most families contact these support groups shortly after receiving a diagnosis (though some families may take years before they are ready to make contact), it suggests that they are seeking information *from other families* to reduce their isolation.

Formal support groups have transformed in recent years from being ‘self-help’ groups (‘so just people talking to people’) to larger, more structured organisations employing large databases, which record medical information (e.g. genotype and phenotype) and family background (e.g. age, education and locality). These databases have become important tools for creating ‘networks’, which allow support group workers to put new families in contact with similar families who live in close proximity.

There is a marked difference between organisation-led support and informal peer support. Respondents talked about accessing verified and trustworthy medical information through support groups, which differed from the practical tips and day-to-day experiences shared online with peers. These differing roles appear to increase the sense of responsibility for people involved in running formal support groups.

In addition to providing verified scientific or clinical information, support groups also provide a space where information about the experience of living with children with 22q11.2 DS can be shared among families. They do this by communicating with parents on social media and through face-to-face events, so that parents can talk about their own experiences with their children and encourage other families to do the same. The support groups are involved in teaching about their rare condition and raising awareness among medical professionals as well as the general public. This sharing may bridge the gaps between clinicians, researchers and parents.

“it can only help our young people…so one of our things is to try and raise awareness but it’s incredibly hard to do” (representative of support group C)

The support group representatives contribute to the support groups in an altruistic manner, with the aim of helping others without necessarily getting paid for it. This also happens on a smaller scale with parents who help each other on the Internet. The code ‘finding the right support’ was an important component of coping for families; some prefer support groups that are more medically focused, while others are more support focused. During interviews, some parents compared the support available, highlighting their reasons for wanting to join one support group over another. Their reasons may include their personal outlook on the condition, available family support, the ages of the children and pertinent issues for their child (e.g. behavioural or heart issues).

…what tends to happen is that they would then stick to the ones that seem to meet their needs more (support group C representative)

[support group representative] does anything she can to help – directs you to somewhere that she feels will help, she won’t take you anywhere that would be unhelpful or that will give you insufficient or inappropriate advice, she will only tell you where to go if she knows where will be best for you (Marianna)

The trust that Marianna has in the support group representative shows the success of the support group in one way and the responsibility of the support group to provide for these families. There is an acceptance that different support groups work in different ways and we can see that they cater to different needs. It is difficult to gauge what a family needs in one particular instance and this changes as the condition progresses.

One example of good practice mentioned and a possible way ahead for support groups to contribute was the production of a consensus document regarding the management of children or adults with 22q11.2 DS (Max Appeal. 2013). This document was produced through the collaboration of support groups, families and professionals who worked together to produce the content.

…you could go through the consensus document and you can tick them [management options] off and everything’s been done and its absolutely hunky dory – the referrals in place and all there, the follow ups are all – you know and everything’s great. (support group D representative)

Increasingly parents are directed to support groups by health care professionals, which is regarded as a positive development.

I think there is a real challenge in sort of…trying to get parents to realise they need to find reliable sources of information […] we’ve tried so hard to get doctors to refer to us (support group A representative)

This quote marks the various networks that a support group manages. It is interesting to note that many of these support groups encourage medical professionals to not only refer to the group but also participate in the group, vetting the information, thereby creating many networks and roles within the support group. This is where the boundaries blur and fade between seeking information, sharing information and giving support.

### Community building via an online platform

All the participants in our study discussed the Internet and social media as a significant aspect of seeking information about 22q11.2 DS. This is exemplified by an avid Internet user in the cohort.

“…the bane of people’s lives he is Doctor Google” (support group D representative and parent of a child with 22q11.2DS)

In this quote, the representative explains how important Google has become to parents—they attribute a sense of authority to it, by referring to it as Doctor Google. It is an essential part of how people seek information and people in support groups rarely go without it.

The Internet allows free and easy access to many sources of information. These factors are reported as important for parents, especially those dealing with many caring responsibilities and complicated appointment schedules.

Parents also reported both negative and positive experiences, and in some cases, information overload, but on the whole, the Internet was regarded as a useful tool. Rather than being passive consumers of information, parents appeared to be discriminating in filtering irrelevant or dubious information. While many described ‘quality information’ as an aspect of information-seeking online, this apparently meant different things to different parents.

P: So I do my own research and I found the Unique page? I think that’s based in the UK also?

R: Yes yes.

and that’s where I found the majority of my information – believe it or not Facebook helped me find more …. when I first joined a year ago there was only like 49 members and now there’s like 70 members (Tania)

In the extract above, we can see one user of the Internet using two very different sources of information. She refers to the ‘Unique page’ which refers to a well-established support group with concise and detailed medical leaflets about various chromosomal disorders including 22q11.2 DS. She also discusses how Facebook, a social media networking site, was a good source to find more information.

In this small-scale study, we noticed that different generations of parents engaged in the Internet in different ways, with parents of younger children more likely to use social media such as Facebook, whereas older parents were more likely to use support group websites or look up scientific journals. This may be due to the rapid increase in the accessibility of the Internet in recent years. However, the data set was too small to know whether this was representative of the population beyond our participants.

From the interviews, social media emerged as playing a major role in how parents use the Internet to seek support rather than just technical information. Two themes that emerged repeatedly in the interviews were ‘sharing’ and ‘community’, which imply that information sharing is embedded in practices of the online community. For instance, there was a strong feeling among parents that sharing improves their experience of coping.

–it varies, it really varies emm mostly it’s trying to support each other, and helping to support our children. Ehh you know and understanding, some people have known about it a lot longer than others. Emm mostly it’s about sharing expectations emm some of it is medical, some if? it’s more ‘hang in there’ sort of thing. (Janet)

The advantage of social media platforms is the way in which support occurs dynamically, enrolling multiple voices. Sharing narratives and experiences evolves spontaneously and in relation to issues and problems as they occur in real time. Communities of sharing specific to 22q11.2 DS offer a combination of technical knowledge, experiential expertise and emotional support. A common ‘unit’ of sharing among parents is the narrative:

…we just tell each other our stories […] we try help each other the best we can…Everyone has shared their own research…shared similarities between our children (Tanya)

The activities of co-creation on social media offer the basis of sharing ‘stories’ of knowledge and expertise. Often, it involves aligning similarities of ‘experiences’ relating to their child’s symptoms, treatment and behaviour. Parents share meaningful strategies of coping with challenging behaviours, or seeking professional support regarding learning difficulties, or practical tips regarding schooling and applying for school support. The co-creation of experiences online provides a knowledge base for developing practical and useful life strategies for coping with 22q11.2 DS.

The Internet was described by two interviewees as ‘shrinking the world’: allowing families with rare conditions to seek each other and form connections. Given that ‘isolation’ is a common issue among families coping with rare conditions, these communities provide opportunities at least to mitigate social isolation. Families coping with a rare condition may be unlikely to find similar families in their local area: through the Internet, they find commonalities with families from across the globe.

it means that people can sort of become? "become friends" in inverted commas with people in different countries […] you know there is someone else out there […] it forms a sense of community… even if you’re up in the middle of the night in the UK, they’ll be someone awake to respond to you (Support group representative)

The term ‘friends’ is evidently a loose description of companionship, but in the world of Facebook, it describes the fleeting relations of people ‘out there’ who are potentially contactable. The Internet has no restriction on time and space which allows online communities to flourish, especially in the ‘shrinking world’ of rare disease.

Support groups have embraced social media and representatives have said that it has increased a sense of community for people dealing with rare conditions. This was an unexpected finding for many which strengthened the support groups in many ways.

I’m not sure we ever envisaged it [social media] would be quite so powerful in bringing people together (Support C group representative)

However, some support groups are wary about the accounts of personal experiences available online, especially since there is such a wide range of stories available.

…there will be people who have either had tremendous success or who have had tremendous disaster. It’s not the people who are bumbling along OK…they haven’t got a story really (support group E representative)

This extract in particular emphasises a phenomenon of Internet story-telling that has been described by many. Some parents may find it hard to relate to stories that seem either too drastic or too positive to help in their own situation. This was not brought up by any of the parents interviewed but was raised by support group representatives who had perhaps supported families with such experiences. They reported families feeling disillusioned or further isolated by reading such accounts, which left them disempowered and unable to connect to people within this community. In our cohort however, parents found themselves able to separate their own story from those written on social media. They could empathise with, and offer support to, families who were struggling.

### Parent empowerment

Parents who were disappointed by their experiences with the medical profession pursued their own avenues of seeking information and support. Some parents were quite explicit in describing a process of acquiring ‘expertise’ in 22q11.2 DS, that is, by seeking and accessing material from a variety of sources. This was mentioned in all eight parent interviews and in four of the seven support group interviews.

Parents described the ways in which acquiring information empowered them to trust their own intuitions and judgements. Living with their child’s condition engenders a sense of ‘lay expertise’ or ‘expertise by experience’ (Bradley 2016). Parents believe that their personal experience puts them in the best position to help by understanding their child’s condition holistically.

This contrasts with the ‘symptom-focused’ approach they feel is the approach somtimes taken by health care professionals. Acquiring lay expertise apparently alters the doctor-patient relationship, particularly when parents take a pro-active approach to their child’s care. This may involve chasing and coordinating multiple appointments and presenting clinicians with new information. But it also creates tensions in the doctor-parent relationship. Parents feel they are being perceived as ‘difficult’ and often need to re-explain the condition and symptoms to new professionals. Some parents describe this process as exhausting.

I was… actually felt like I was educating him. And he was then talking down to me, not knowing I was a nurse and this and that but very unprofessional, talking down to me – telling me that nothing was wrong with him, and to treat him like a normal person and I do. Like he was just making me feel like I wasn’t (1) being the parent that I should. And I shouldn’t treat my son different no matter what… (Tanya)

In developing lay expertise, parents become ‘authoritative’ in their own knowledge; which they feel is different from the information they can glean from clinical appointments. Using this information, they shared the feeling that they are better prepared to meet the challenges of living with 22q11.2 DS.

## Discussion and conclusion

The interviews with both parents and support group representatives highlighted specific aspects of how a support system works that had not been anticipated.

Our interviews correspond to van den Bree et al.’s (2013) work concerning the sources of information families may draw on. A number of parents sought alternative information sources to clinicians, as van den Bree et al. describes. From our analysis, this diversion from traditional clinical settings seems to come about because parents experience clinical sources of information as insufficient—or insufficiently relevant to their situation. The information from clinicians is often given without support, and this makes the information more difficult to absorb and cope with. The feeling of ‘medical abandonment’ causes parents to begin the process of becoming ‘expert’ by collecting and sharing information in a process that brings together different types of expertise—medical or scientific—with practical information and experiences from fellow parents, and the understanding of their own personal journey.

Our study was an inductive one and did not use a particular information-seeking model as a framework for analysing the data.

**Fig. 1:**
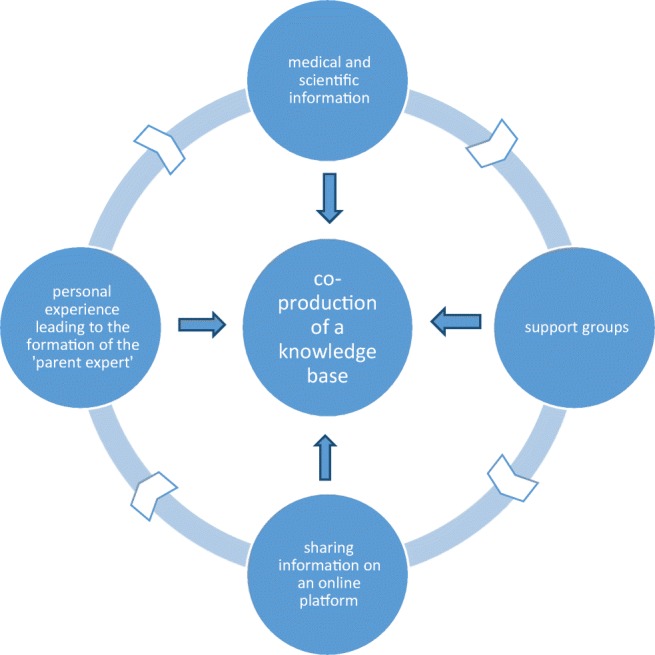
Co-production of a knowledge base

Using the results detailed above, Fig. 1 was constructed as a possible model of how the 22q11.2 DS community builds a knowledge base. This is what was induced from the discussions with support groups and families. The process of information seeking and sharing is not a linear one and a number of factors contribute to what we have called a ‘co-production of a knowledge base’.

Our findings indicate that the Internet, social media and support groups are instrumental in the building of this knowledge base. Studies have previously suggested that information seeking usually happens through conventional online sources (Rains 2008). However, our results indicate that social media provide a valid source of information for these families. This could be because a number of support groups are now based online, with easily accessible pages on Facebook. It is clear that the fears of many health professionals, and some researchers, about unverified sources of information online (Ziebland and Wyke 2012) are not really regarded as a risk by many caregivers affected by the condition in our cohort.

One can observe a change in what these communities go through. In the past, patients were for the most part, submissive ‘consumers’, allowing clinicians to show a level of paternalism, and an unwillingness to share what they would deem ‘extra’ information. Now a patient approaches a clinician with knowledge gleaned from these online communities with the idea of mutual education and production of knowledge.

Multiple Internet-based platforms have developed rapidly in the past decade. It has become much simpler to seek information online so that it is now accessible to a much wider audience (Chung J.E 2014, Fox 2012). Parents reported the Internet as being easy to access; they find the accessibility of the free services available for both gathering information and for connecting with other families particularly valuable. Less traditional sources are sought when the traditional route yields less information (Mustafa et al. 2015, Ziebland and Wyke 2012, Colineau and Paris 2010, Rains 2008).

Support groups were described as an optimal source of quality information. They combined the expertise of professionals with the use of readily accessible language (avoiding scientific jargon). Parents found this extremely helpful, especially in the first few weeks following a diagnosis when they started learning about the condition. This level of expertise has not previously been explored in the academic literature.

The expertise of the broader patient and family community enables more cooperation between patients and providers, with support groups enabling the flow of that information by developing information leaflets for and with clinicians, organising joint patient-provider conferences and opportunities for participation in research. This co-production creates what has been termed ‘distributed knowledge’ that eventually becomes ‘authoritative knowledge’. Parents, by sharing the knowledge that they have gathered by living with and learning from their children who have 22q11.2 DS (termed distributed knowledge), become authorities in that information, having the power to share it and help other families (authoritative knowledge). Similar findings have been experienced in studies of ALS (Kazmer et al. 2014) and Hyperemesis Gravidarum (Lowe et al. 2009) as well as other rare genetic and chronic diseases (Van der Eijk M et al. 2013). Vicari and Cappai (2016) refer to this as the logic of connective action; these digital mechanisms help to overcome information-seeking obstacles and allow the development of different forms of engagement which, as represented in our diagram above, is not a one-way channel.

Being active participants in sharing information can empower these individuals and communities, as evident in the interviews. The parents themselves found that sharing anecdotes and information with other families developed a sense of community that met a previously unrecognised need and helped them to cope better. This was an unexpected finding, even a surprise, for many.

Discussion with support groups revealed that a large component of their work involves ensuring that parents have access to accurate information and emotional support. Support groups work with parents and scientists to encourage participation in research, thereby ultimately increasing the availability of knowledge about 22q11.2 DS. This has been seen in the recent example of the Consensus Document published by ‘Max Appeal!’ in 2013, which professionals are encouraged to use to guide the care of patients of 22q11.3 DS (Max Appeal 2013).

This community sharing of expertise comes with risks, including that low-quality and unverifiable information may be disseminated. This risk seems to be a particular concern to individuals involved in running support groups. The personal narratives available through these sites can involve a broad range of contrasting experiences, some reporting great sadness and difficulties while others recount tales of good practice and problems that have been managed well or avoided. The accounts provided will often not be representative of the range of possible family experiences but be biased to the extremes, which can lead to anxiety on the one hand or false hopes on the other (Ziebland and Wyke 2012, Fox 2012).

Comparable processes to the figure developed above have been described in Internet-based medical health interactions in relation to cancer, diabetes and other chronic illnesses (Hilliard et al. 2015, Van der Eijk et al. 2013); our data correspond closely to these reports.

### Practice implications

Parents desire more support from clinical services, especially in regard to coping strategies. There is a need for more psychotherapeutic counselling for parents of the complicated condition of 22q11.2 DS, which has been argued as the way forward for genetic services (Austin et al. 2014). This correlates with findings from other researchers (Huff 2015, Dimond 2010, Plumridge et al. 2010). Our findings suggest that more input from a genetic counsellor at the diagnosis stage would be welcome. Genetic counsellors have a unique skill set grounded in both genetic information and Rogerian counselling skills. Austin advocates for more of the counselling skills that are learned in training to be implemented in clinic, as the way to really connect with patients. Our data supports this from the perspective of parents.

Clinicians are often described as being wary of the Internet as websites may have potential unverified information. Van der Eijk et al. (2013) emphasise the need to make more use of online health communities to deliver communication and support coordination for these families as well as working with support groups to direct patients towards quality information.

### Limitations and further research

The strength of this study has been its exploration of the role of support groups that is beginning to acquire in bringing professionals closer to understanding the condition as well as addressing the personal and emotional needs of parents.

It is important to note that the small sample size of the qualitative interviews prevents us drawing conclusions about possible differences in access to health services, support groups or the Internet among different communities, ethnicities and countries. This merits further research.

The ‘digital divide’ described by many in Internet communication research was not observed in our data. This is probably because participants were recruited through online methods and we were therefore reaching those participants who were already using these digital information-seeking tools (Lustria et al. 2011). It will be important to extend research in this area to include people who are not digitally enabled, to see how they fare in a world where sources of information are increasingly moving online.

Recruitment was sought through support group representatives and this could have biased the results. As our findings indicated, people who become members of support groups feel a strong sense of loyalty to their chosen support group and are therefore more likely to praise the efforts and the strategy of that support group. Recruiting through NHS clinics could have resulted in a reduction of such biases.

The findings reveal a synergy between the different stakeholders in 22q11.2 DS. The support groups are an essential part of the chain that links parents to clinicians, information and other parents. Further research into these organisations and their goals may help professionals better understand the support group role in patient care. Such studies have already revealed positive impacts of support groups in terms of helping families with coping.

### Conclusion

Information-seeking behaviour in parents of children with 22q11.2 DS is complex. Information seeking cannot be separated from the intricacies of peer support where parents can both seek and provide information. Social media and online support have changed parents’ experiences and have helped connections to be made in situations that might otherwise lead to isolation. Together, these multiple interactions contribute to a knowledge base constituted from multiple levels of expertise. It also appears that sharing and altruism improve the experiences and coping capacities of families.

We have developed a model, based on our analysis of interviews with support groups and parents, of how a base of knowledge is gathered about 22q11.2 DS and how information is shared and sought. These connections have resulted in changes in the management and outlook of 22q11.2 DS over the past few years, and we have discussed ways on how we could use this knowledge base in clinical practice to aid patient care and support.
